# Supplementary material for: A controlled study to assess the effects of a Fast Track (FT) service delivery model among stable HIV patients in Lusaka Zambia
Source: PLOS Glob Public Health. 2022 Aug 3;2(8):e0000108. doi: 10.1371/journal.pgph.0000108 (PMC10021658; doi:10.1371/journal.pgph.0000108)
Supplement: Attachment — Submitted filename: ft_review_response-29mar2022.pdf [file pgph.0000108.s003.pdf]

CIDRZ

for a healthy Zambia

Joel Msafiri Francis, MD, MS, PhD
Academic Editor
PLOS Global Public Health

Dear Dr. Francis,

Many thanks for reviewing our manuscript (PGPH-D-21-00138) titled "Effects of a Fast Track (FT) service delivery model among stable HIV patients in Lusaka Zambia".

On behalf of my colleagues in Zambia and the United States, I would like to thank you and the reviewers for the feedback. We have reviewed each comment carefully and have made the changes noted below, which we believe have further strengthened the manuscript. We have enclosed two versions of the revised manuscript in Word (.docx)—one with changes highlighted under "track changes" and a second "clean" version with all changes accepted.

In response to the CONSORT request, we kindly request that we review the proposed changes in the text. Whilst this was part of a larger randomized trial, the Fast track intervention and control sites were purposively chosen- sites needed to be large, have space to implement Fast Track and be within a certain geographic area for access- and thus it does not qualify as a true 'randomised trial' and should not be reported under the CONSORT guidelines. We believe this has been adequately described in our submission. However, we are happy to provide additional information or make further edits if required.

We believe this revised version of the manuscript addresses all of your other concerns.

Please find here our point-by-point responses to reviewer comments, organized by headings taken from your February 6, 2022 email (also, please note that for ease of reference the lines below refer to the "clean" version of the revised manuscript):

We would like to thank the reviewers and editors, again, for assistance in improving this manuscript. We hope that it is a better fit for PLOS Global Public Health.

Kind Regards,
Jake

Jake M. Pry, PhD, MPH
Centre for Infectious Disease Research in Zambia (CIDRZ)
jake.pry@cidrz.org | jmpry@ucdavis.edu
mobile: +1-936-661-6885 | +260 96 3088750

Reviewer #1: I am afraid that the revisions do not seem to be complete or to address all of the points yet. I am happy about the data not being available due to this being MOH data and so this is not an issue.

My major issue is still with the design and the assumptions that the authors make regarding the comparability of the clinics in the two groups. Whilst the design has become clearer- that from a pool of 28 clinics 16 were randomly selected for DSD interventions of which 2 were selected to have FT (the subject of this paper). From what I can understand is that of 16 with DSD interventions 5 were selected to be FT controls (but were therefore not full SoC clinics as they had some DSD?).

Response: Thank you for this thoughtful comment. We have adjusted the narrative in the abstract, and methods sections to clarify that control faciltiies as clinics at which there was no study involvement and in which no formalized differentiated service delivery model were being delivered.

Abstract: “We selected a systematic sample of patients from study sites, including people enrolled in FT at the intervention sites and individuals enrolled at the control sites (representing standard of care in which no differentiated service delivery models were being considered) meeting FT eligibility criteria.”

Methods section: “As a part of a cluster randomized study design (ClinicalTrials.gov: NCT02776254), we conducted a controlled study to compare a FT service delivery model to the current standard of care (SoC) represented by comparable facilities at which no differentiated services outside of those recommended/mandated by the Ministry of Health were implemented, among stable patients on ART”

Since this does appear to be a cluster randomised trial its reporting does not follow the standard consort guidelines in that the abstract and title do not include information that this is a randomised trial, we do not have information about the randomisation process nor blinding etc. A consort checklist should be provided.

Response: We have responsed as able to this request given limitations of data availability.

Since the number of clusters is so small and uneven (there really need to be at least 6 per arm to have any confidence in this) there are likely to be many existing biases that would not be overcome by randomisation and therefore assumptions about these are incorrect. This needs to be discussed more in the discussion.

Response: We have added narrative in discussion section in the limitations as follows: “Finally, we observed heterogeneity in the outcome of late ART pick up by facility, evidenced in table 3, which may limit generalizability however, we describe underlying intervention and control participants and present adjusted analyses, with random effects at the facility level, to mitigate differences across facilities.

The clinics are still named- which contravenes their confidentiality as research participants

Reponse: We have de-identified the clinics.

I note that the authors have chosen to remove the qualitative section to another paper but the abstract and methods still imply that this is a mixed methods study and so needs further editing (methods line 173/4, abstract needs revision throughout)

Response: Thank you, we have omitted language detailing qualitative methods.

The abstract does not now fit the details in the main body of the article and needs a thorough revision to match the methods etc.

Response: We have thoroughly revised the methods section of the abstract to better represent the study.

Abstract (methods): "We conducted a controlled mixed-methods study (only quantitative data presented here) to compare a "Fast Track" (FT) service delivery model to the current standard of care among stable patients on ART. In the parent study, 16 purposively selected health facilities (HF) were randomized to provide one of four different DSD models. For this analysis, we included the 2 sites assigned to FT and 5 randomly selected standard of care sites. We selected a systematic sample of patients from study sites, including people enrolled in FT at the intervention sites and individuals enrolled at the control sites (representing standard of care in which no differentiated service delivery models were being considered) meeting FT eligibility criteria. We used programmatic data from electronic and paper medical records to compare missed drug pick up visits and medication possession ratio (MPR) amongst patients enrolled in FT compared to those theoretically eligible from control/standard of care sites."

Reviewer #2: Thank you for the opportunity to review this manuscript, which addresses an important question regarding the impact of the Fast Track differentiated HIV service delivery models on outcomes such as missed visits and retention in care. While the topic is of high interest, especially the deep dive into programmatic data, the manuscript itself is not ready for publication. In particular, removing the qualitative data from the methods and results sections, but leaving them in the abstract, introduction and discussion sections is problematic. A more minor point is that manuscript needs a close edit – there are a few typos, grammatical errors, and misspellings.

Abstract:

The abstract does not accurately describe the study and is inconsistent with the rest of the paper. The methods section is confusing (see below) making the conclusions section hard to interpret. A few additional notes:

Row 51: The paper as currently presented is not a mixed methods study, so this should be re-edited

Response: We have revised narrative around mixed effects in the astract and omitted qualitative methods in the methods section to address this feedback.

Abstract (methods): "We conducted a controlled mixed-methods study (only quantitative data presented here) to compare a "Fast Track" (FT) service delivery model to the current standard of care among stable patients on ART. In the parent study, 16 purposively selected health facilities (HF) were randomized to provide one of four different DSD models. For this analysis, we included the 2 sites assigned to FT and 5 randomly selected standard of care sites. We selected a systematic sample of patients from study sites, including people enrolled in FT at the intervention sites and individuals enrolled at the control sites (representing standard of care in which no differentiated service delivery models were being considered) meeting FT eligibility criteria. We used programmatic data from electronic and paper medical records to compare missed drug pick up visits and medication possession ratio (MPR) amongst patients enrolled in FT compared to those theoretically eligible from control/standard of care sites."

Row 52: The descriptions of the sampling and data analysis are very confusing. I do not want to tell the authors what to write, but here is an example of something that seems easier to understand:

Response: In the parent study, 16 purposively selected health facilities (HF) were randomized to provide one of four different DSD models. For this analysis, we used the 2 sites assigned to FT and 5 randomly selected standard of care sites. We then selected a systematic sample of patients from each site, including people enrolled in FT at the FT intervention sites and those eligible for but not enrolled in FT at the control sites. We used programmatic data from electronic and paper medical records to compare missed visits, retention in care and MPR amongst pts enrolled in FT vs those receiving standard of care.

Row 54-56: Since the qualitative part of the study has been edited out, you should not be referring to IDIs and FGDs in the methods section.

Response: We have omitted language detailing qualitative methods in the methods section.

Row 67: This point applies to the paper in general, which is the need to define the terms: missed medication pickup appointment vs. retention in care. It will also be important to clarify if the study tracked other types of appointments. Could patients have missed a drug-pickup appointment (at the pharmacy, for example) but not a clinical appointment?

Response: We have limted reference to "retention" and clarified outcome where appropriate as late ART pick-up. It is possible that other types of vists could occur however, we have found that pharmacy visits are the most robust indicator of clinic visit and so have defaulted to a parsimonious outcome limited to pharmacy visit.

Introduction:

It would be very helpful if the introduction provided more background about the FT model in Zambia and a clearer description of how it differs from standard of care (SoC). For example, I infer from the rest of the article (including the conclusion!) that 3-multi-month dispensing (3MMD) of ART is provided in both FT and SoC, but this is not clearly described. In particular, FT is presented as being a more efficient model, but appears to involve more time and space than SoC. It might also improve clarity to briefly describe the parent study here, rather than in the methods section.

Response: Thank you for this feedback. We have added language describing the standard of care and ART refill in the introduction as follows: "The standard of care in Zambia at the time of study initiation included a one-month ART refill schedule, a first-come first-served single, common queue service point with patient volume in excess of clinic design and staffing."

A few additional notes:

Rows 82-84: Minor point: These sentences and their references refer only to the experience in Zambia, which is not universal. I recommend that the authors make it clearer that they refer to the Zambia experience.

Response: We have revised given feedback as follows: "By 2017, patient volumes at facilities had grown rapidly and waiting times for clinical services reaching 3-4 hours (or longer) per visit were commonplace in Zambia. For patients already on treatment, negative experiences accessing care has emerged as a critical factor in undermining the sustainability of treatment. The standard of care in Zambia at the time of study initiation included a one-month ART refill schedule, a first-come first-served single, common queue service point with patient volume in excess of clinic design and staffing. Reducing waiting time for HIV services and improving HIV clinic visit experience represent opportunities to optimize epidemic control in resource-limited settings, like Zambia."

Row 89-90: Minor point: DSD is intended to improve quality and client satisfaction, not just efficiency.

Response:

Rows 91-92: While it is true that this paper provides more rigorous statistical analysis of programmatic data than I have seen elsewhere, the assertion that "the impact of DSD models on retention and other outcomes has not previously been documented with rigorous studies" is not correct. Would either describe the growing literature on the impact of DSD in the introduction or rephrase this statement if length constraints prevent a discussion in the intro section.

Response: Thank you for this important point, we have updated language consistent with the current gap given the recent work in this area as follows: "Although these DSD models are exciting innovations that, in theory, meet public health needs, impact of an expedited service delivery model on pharmacy visits in Zambia has not been well documented – a critical addition given the inclusion of the FT model in Zambia's differentiated service delivery agenda."

Row 94: "form" should be "from"

Response: Thank you, we have corrected to "from".

Row 105: Remove reference to qualitative data collection and the term "mixed methods"

Response: We have removed all reference to mixed methods.

Methods:

This section is very hard to understand. Key information is out of order, some points are repeated, and others are omitted. Topics are mingled in ways that are not helpful. Study design is described twice (lines 108 and 164) and remains confusing, as does the extent to which the two arms were exposed to MMD and visit spacing during the study period. Site selection is described in lines 126ff and again in line 167ff. There are references to changing drug dispensing intervals in the introduction and briefly in the methods (line 123) but no details; there is also a note in the discussion that the design of the model offered at SoC sites changed during the study, but this is not mentioned earlier. References to the qualitative study elements are mostly but not entirely removed. Other issues include but are not limited to:

Response: Thank you for this feedback – we have re-written large portions of this section removing redundance, standardizing language, and clarifying the specific well outlined below.

Although both FT and SoC are described in the section starting on line 150 ff, it would be helpful to clarify the differences earlier in the paper, perhaps even in the intro section.

Modifying Figure 1 to highlight the key similarities and differences both from patient flow and staffing/resources perspectives might be efficient. Patient services: more streamlined flow in FT, more frequent clinical visits in SoC, 3MMD in both, etc. Resources: staffing, space, etc.

Response: We have added the following in the introduction to improve contextualization of the study: "The standard of care in Zambia, at the time of study initiation, included a one-month ART refill schedule, a first-come first-served single, common service point/queue with patient volume in excess of clinic design and staffing. We assessed a differentiated service delivery (DSD) model aiming to reducing waiting time for HIV services and fewer pharmacy visits compared to standard of care in Zambia."

As above, my understanding is that 3-MMD was provided to patients in both arms, that the FT arm had clinical visits every 6 months and the SoC arm had clinical visits every 3 months. Thus, MMD and visit spacing should not be conflated.

Response: We have edited narrative as follows in response to feedback: "The standard of care in Zambia at the time of study initiation included a mean one-month ART refill schedule, (though 3-month refills had been recommended for stable patients in alignment with World Health Organization standards since 2014), clinic visits operating on a first-come first-served schedule through a single, common queue service point with patient volume often in excess of clinic design and staffing. Reducing waiting time for HIV services and improving HIV clinic visit experience represent opportunities to optimize epidemic control in resource-limited settings, like Zambia."

When describing the parent study, it would be helpful to provide more info about site selection – for example, to characterize whether the 16 CIDRZ-supported HF are systematically different in any way from the 12 other HF in the province

Response: It is possible that data availability differs across sites, otherwise all facilities are similar and uphold the Ministry of Health HIV care delivery standards.

Line 114: there is no definition of the primary study outcome "retention to care"

Response: We have omitted language referring to primary outcome as retention and revised to refer to late for ART pick-up.

As above, does missing a drug pickup appointment necessarily mean not being retained in care? In row 111, there is reference to tracking clinical appointments, but I don't see these data in the results section. If they were one and the same at either FT or SoC sites (i.e., if meds were dispensed at the clinical visit), I recommend explaining this.

Response: Clinical visits in the SoC were conducted on an ad hoc basis or every 3-6 months per Consolidated HIV Care Guidelines 2013

Line 110, 124, 133, 142-145, 184: remove references to qualitative data and/or the term "mixed methods"

Response: We have omitted reference to qualitative data.

Results:

Visit spacing data are difficult to understand without a description of the intervention. There is an implication here and in the discussion section that appointment intervals and multi-month dispensing intervals were varied in some way, but no description of how this worked or how it affected data collection/analysis

Response: We have revised results sub-section visit spacing as follows: "During the study period the conventional refill was extended from 1-month to 3-months resulting is a mix of individuals in the control faculties receiving 1-month and 3-month refills."

Discussion:

I may have missed this, but if the SoC patients had more appointments in the study timeframe, wouldn't that increase their cumulative probability of having missed/been late for at least one? Doesn't that introduce ascertainment bias?

Response: This is part of the appeal of the extended refill associated with the FT model and indeed, the shift during the study period the standard of care shifted to a median of 3-month refills, more appointments corresponds to more opportunities to be without ART. We have added supplemental KM estiamtes by ART refill interval:

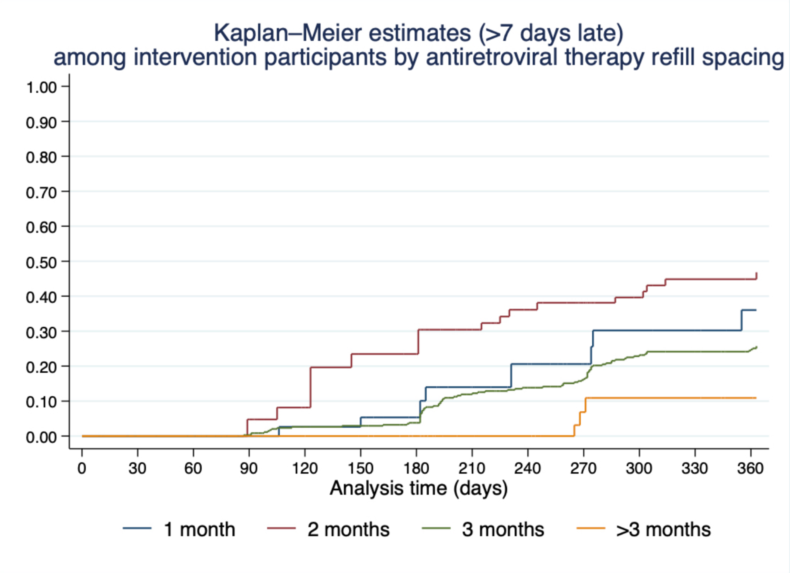

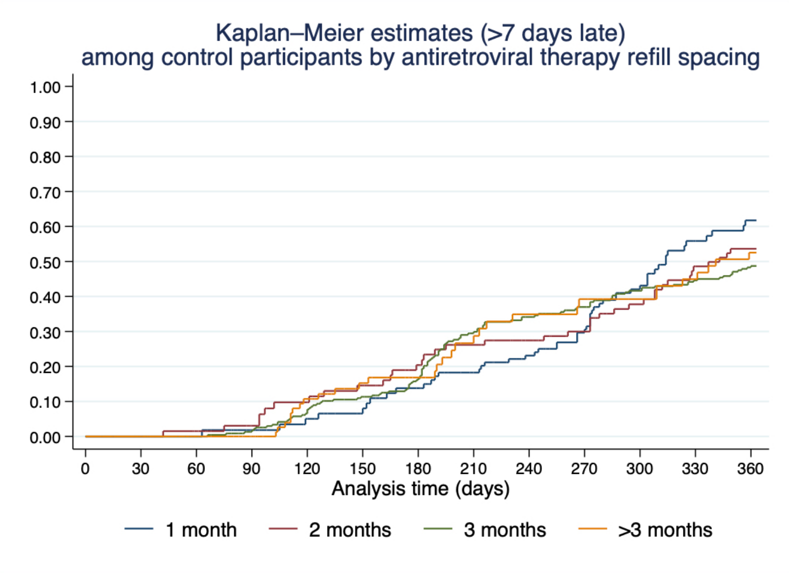

I do not think the authors have entirely resolved the issue of potential selection bias flagged by earlier reviewers, so this should certainly be discussed in this section, if only to reiterate the data analysis techniques that were used to mitigate.

Response: We have added a supplemental figures outlining the heterogeneity in the control group compared to intervention group and the increased incidence of late for pick up among the control group compared to intervention group regardless of refill interval indicating that ART refill interval alone is likely not the reason for improve ART pick-up among intervention particpants.

As above, it's difficult to interpret statements like the following without more detail: "Examination of the effect of FT across strata of prescription length practice revealed an interaction where longer duration and FT alone had little effect on retention whereas both together had far larger effects."

Response: We have modified narrative in response to feedback as follows: "Examination of the effect of FT across strata of prescription length revealed an interaction where longer duration and FT alone had little effect on late ART pick up whereas both together had far larger effects (Figure 5)."

Line 336: Should be "drug pick-up" not "drug pick"

Response: We have revised to "ART pick-up".

The description of other DSD models in Zambia (line 341) should probably be in the introduction or the settings section, not in the discussion

Response: We have acknowledged and cited other DSD models in methods.

Line 345: Would not refer to the findings of the parent study unless you are going to say what they are and cite.

Response: We have omitted reference to parent study and added references to support statement: “Findings here as well as those published by others regarding DSD models in sub-Saharan Africa, suggest that an array of differentiated care options may improve retention through reduced late ART pick up and lend to optimized HIV care in Zambia and beyond”

Line 356: remove reference to qualitative data, since not included in the study

Response: Reference to qualitative data has been omitted

Line 358: Would remove this whole paragraph – you shouldn’t cite data that is not presented in the methods and results sections

Response: We have omitted in accordance to feedback

Line 365-368 (“Whilst this model…”): Would remove, or cite data to support

Response: We have omitted in accordance to feedback

Lines 365-377 This whole paragraph is vague… not sure it adds anything critical.

Response: We have abbreviated and tightened to convey the mix of challenges and potential for differentiatied service delivery models as follows: “As ART programs continue to grow, and the burden on the health system continues to swell, identifying cost effective, sustainable solutions for decongesting clinics and improving efficiencies remains key. Additionally, ensuring that these DSD options are responsive and flexible to accommodate the neccessary changes to meet patients where they are in their care journey including potential drift in and out of different models, changing social circumstances, stages of clinical well-being, and other individual needs. FT, whilst not able to address all barriers, is definitely a viable, cost-effective model that over the relative short term has shown to be successful at retaining patients and improving adherence.”

Lines 380-382: As above, this is the first time that programmatic shifts in refill intervals are mentioned

Response: We have added language in the introduction and methods to detail the prgramatic shift in ART refill during the study window.

Line 384: (“Differentiated service delivery models such as FT likely have highest value for retention improvement/optimization and may be most applicable in the urban settings where clinic congestion can be a particularly difficult challenge for patients.”) Not sure why this is in the limitations section?

Response: We have omitted per feedback.

Line 387: Would remove the reference to viral suppression being an intended outcome, or add the point to both methods section and results section and explain…

Response: We have omitted per feedback.

Line 387-389: remove this reference to qualitative data that is not in the results section

Response: We have omitted per feedback.

Implications: remove reference to qualitative data.

Response: We have omitted the qualitative data findings from implications.

End of comments:

We would like to thank the reviewers and editors, again, for assistance in improving this manuscript. We hope that it is a better fit for PLOS Global Public Health.

Kind Regards,
Jake

Jake M. Pry, PhD, MPH
Epidemiologist
Centre for Infectious Disease Research Zambia
jake.pry@cidrz.org | +260 96 3088750 | +1 936-661-6885
