# Supplementary material for: A controlled study to assess the effects of a Fast Track (FT) service delivery model among stable HIV patients in Lusaka Zambia
Source: PLOS Glob Public Health. 2022 Aug 3;2(8):e0000108. doi: 10.1371/journal.pgph.0000108 (PMC10021658; doi:10.1371/journal.pgph.0000108)
Supplement: Attachment — Submitted filename: ft_review_response-15jun2022_final.pdf [file pgph.0000108.s004.pdf]

CIDRZ

for a healthy Zambia

Joel Msafiri Francis, MD, MS, PhD
Academic Editor
PLOS Global Public Health

Dear Dr. Francis,

Many thanks for reviewing our manuscript (PGPH-D-21-00138) titled "Effects of a Fast Track (FT) service delivery model among stable HIV patients in Lusaka Zambia".

On behalf of my colleagues in Zambia and the United States, I would like to thank you and the reviewers for the feedback. We have reviewed each comment carefully and have made the changes noted below, which we believe have further strengthened the manuscript. We have enclosed two versions of the revised manuscript in Word (.docx)—one with changes highlighted under "track changes" and a second "clean" version with all changes accepted.

We have made every effort to address the points outlined by the reviewer and believe this revised version of the manuscript addresses all concerns raised over this year of review.

Please find here our point-by-point responses to reviewer comments, organized by headings taken from your April 28, 2022 feedback (also, please note that for ease of reference the lines below refer to the "clean" version of the revised manuscript):

****begin review feedback****

Centre for Infectious Disease Research in Zambia
Plot # 34620 Corner of Lukasu & Danny Pule Roads, Mass Media, P.O. Box 34681 Lusaka, Zambia 10101
Reception: +260 211 242 257 - 63
Email: info@cidrz.org @cidrzinfo https://www.facebook.com/CIDRZ www.cidrz.org

Having re-reviewed the manuscript it is now becoming slightly clearer in terms of methods but still has some issues- see below.

I would agree that since this is not a randomised trial (this is now clearer- thankyou) then the consort statement does not make sense, but the methods are still very confusing and repetitive as well as contradictory. The whole methods section could do with being rewritten from scratch being transparent about what was actually done or not done. Using a standard reporting structure would help with this.

Response: Thank you for this comment. We have overhauled the methods section with a complete re-write of the settings/population, recruitment and design sub-sections. We have removed reference to randomization as it was nested within a larger trial and does not, in our view, materially influence the study results. We consider this therefore a study where we examine the experience of two facilities receiving an intervention (introduction of fast track) vs. five facilities which did not, and which were selected based on similarity in size and level to the two intervention facilities, in theory to minimize between cluster variability.

<u>Introduction:</u>
Line 1 HIV is now missing and so the opening sentence does not make sense unless you know this is about HIV

Response: Thank you. We have revised to read as follows: "To reach HIV epidemic control, countries must offer reliable, high-quality, long-term antiretroviral therapy (ART) to those persons on treatment while continuing to absorb new patients accessing care and treatment services."

Line 123- I think the previous version was better as there were several outcomes assessed.

Response: Thank you, we have detailed the outcomes as in previous versions as follows: "We examined the effect of the FT model on incidence of being late (>7 days, >14 days, >28 days, and >90 days) for next pharmacy appointment, medication possession ratio, and time to return after missing or being >28 days late for next pharmacy appointment."

<u>Methods:</u>
Line 138 and line 150: I still do not believe that this was a random selection- you state that they were chosen based on geography, size etc to be the best possible counterfactual in line

Response: The random selection of intervention and control facilities was accomplished among a total of 23 comparable high volume, urban HIV care facilities. We have re-written this section to improve clarity.

138- in this is the case then the choice was NOT random. In line 150 you then say there were 12 sites remaining and you randomly picked 5 of them? Were they chosen or randomly selected? If randomly selected then how? Did you stick a pin in a map or use a random number generator etc etc? I really feel you did not randomly chose them you chose those that provided the best counterfactual- if so, then say so

Response: Although the sites were selected through random number generation, again this procedure was done as a part of a larger study and the selection of the sites is not important for the interpretation here, in our view, and removing the references to random selection of the sites will ease interpretation and make the process more transparent. We now simply say we have five control sites selected based on similarity in size and health-systems level to the two intervention facilities.

Line 186 onwards seems to repeat what was said earlier but again to contradict it slightly. I would suggest you could combine these sections and ensure that they state the same thing.

Response: We have revised the entirety of the methods section to improve consistency and clarity.

Line 158 and line 190 again seem to contradict each other- in line 158 you say convenience sampling was used but in line 190 say that random sampling was used.

Response: In this revision, we dropped discussion of how facilities were sampled. We now simply refer to the systematic sampling of individuals at the facility for inclusion in the study throughout the paper.

The outcome measures are not described clearly and would benefit from being defined clearly.

Response: We added a sub-section to the methods "Outcomes"

Results:
Line 283- this should have been better explained in the methods section as it is not a result per se. In the methods you need to state the standard of care and any changes that occurred during the study. You can this discuss this in the discussion section.

Response: We have added a sub-section in the methods to describe the standard of care (control). Additioanlly, the modified visit spacing is dicussed in the results section as follows: "Though part of the intervention package, the median time from visit date to next pharmacy appointment (visit space) between intervention and control sites were similar at 91 days (IQR:89-91) and 91 days (IQR: 63-91), respectively. During the study period the conventional refill was extended from 1-month to 3-months resulting is a mix of individuals in the control faculties receiving 1-month and 3-month refills."

Discussion:
Line 312 "incidence of 90 days late for ART" is a difficult phrase. I think it is referring to the cumulative incidence of being more that 90 days late for ART pick up over the entire period? I wonder if you can either amend the language or come up with an easier terminology? It would be good to introduce the outcome measures more clearly in the methods section and then to use the same terminology throughout

CIDRZ *for a healthy Zambia*

Response: We have added a sub-section for outcomes in the methods section to improve clarity.

"Outcomes
The primary outcome was first incident of being >7 days late for a pharmacy appointment. We also assessed the first incident of being >14 days, >28 days, and >90 days late for pharmacy appointment. Additionally, we calculated medication possession ratio (MPR) based of pharmacy refill data and developed a binary variable of at least 90% medication possession or <90% medication possession across the study period. Finally, we assessed time until return after an incidence of being >28 days late for a pharmacy appointment."

****end of feedback response****

We would like to thank the reviewers and editors, again, for assistance in improving this manuscript. We hope that it is a better fit for PLOS Global Public Health.

Kind Regards,

Jake M. Pry, PhD, MPH
Centre for Infectious Disease Research in Zambia (CIDRZ)
jake.pry@cidrz.org | jmpry@ucdavis.edu
mobile: +1-936-661-6885 | +260 96 3088750

Centre for Infectious Disease Research in Zambia
Plot # 34620 Corner of Lukasu & Danny Pule Roads, Mass Media, P.O. Box 34681 Lusaka, Zambia 10101
Reception: +260 211 242 257 - 63
Email: info@cidrz.org @cidrzinfo https://www.facebook.com/CIDRZ www.cidrz.org
