# Supplementary material for: A controlled study to assess the effects of a Fast Track (FT) service delivery model among stable HIV patients in Lusaka Zambia
Source: PLOS Glob Public Health. 2022 Aug 3;2(8):e0000108. doi: 10.1371/journal.pgph.0000108 (PMC10021658; doi:10.1371/journal.pgph.0000108)
Supplement: Attachment — Submitted filename: ft_review_response-08nov2021.docx [file pgph.0000108.s002.docx]

**Centre for Infectious Disease Research Zambia
Implementation Science Unit**

Jake M. Pry, PhD, MPH

Research Scientist – Epidemiologist

[jake.pry@cidrz.org](mailto:jake.pry@cidrz.org) | +260 96 3088750 | +1-936-661-6885

Joel Msafiri Francis, MD, MS, PhD

Academic Editor

PLOS Global Public Health

Dear Dr. Francis,

Many thanks for reviewing our manuscript (PGPH-D-21-00138) titled “Effects of a Fast Track (FT) service delivery model among stable HIV patients in Lusaka Zambia”.

On behalf of my colleagues in Zambia and the United States, I wanted to thank you and the reviewers for your feedback. We also appreciate Reviewer #2’s positive feedback that “This [manuscript] is an excellent study and definitely fills a needed evidence gap in the HIV response…”. We have reviewed each comment carefully and have made the changes noted below, which we believe have substantially strengthened the manuscript. We have enclosed two versions of the revised manuscript in Word (.docx)—one with changes highlighted under “track changes” and a second “clean” version with all changes accepted.

We believe this revised version of the manuscript addresses all your concerns.

Please find here our point-by-point responses to reviewer comments, organized by headings taken from your March 20, 2020 email (also, please note that for ease of reference the lines below refer to the “clean” version of the revised manuscript):

Reviewer #1: Many thanks for this interesting paper on fast track ART delivery. The area of how to improve patient retention and viral suppression with DSD models is very critical. I do however have some major concerns with the paper as it is currently written

Methods

1. The methodology is extremely unclear. The abstract describes this as a controlled study whereas the methods section in the main paper presents this as part of a cluster-randomized trial NCT02776254. While this trial does mention fast track in the description on clinical trials, what is being described here is not cluster randomized as far as I can see. The description in the methods, states that sites were purposively sampled with the “best possible counterfactuals”- however it is unclear how the sites were actually sampled and why only 2 intervention sites were selected and 5 control sites? In the description of the larger study it states that from 28 sites 13 were selected to be intervention sites (It seems that it should be 14, and note that the primary paper from the trial states that 10 were intervention and control sites?). It then suggests that the 2 FT intervention sites and the 5 FT control sites were selected from the intervention sites. Is this correct? If so they presumably had other DSD interventions that are not mentioned here- could these have biased the outcomes of this comparison?

Response: This is an important note and authors have revised the narrative in several areas to improve clarity and accuracy of methods employed, especially around recruitment.

2. Although unclear whether this is a cluster-randomized study or not the “participants” in this study are actually the clinics as it is these that were allocated into FT intervention or control. As such the flow diagram shown as figure 2 does not match this allocation schema. Furthermore, in the results section you name the clinics. As participants would never be named it is generally not acceptable to name clusters and I would suggest they should only be identified by a study number. However, since they are named they do not seem to be “best possible counterfactuals”. I am concerned around the potential biases from extremely different clinics in the intervention and control arms. I cannot see any data presented to demonstrate that the clinics in the 2 arms were similar with respect to numbers of patients in care, numbers of staff, facilities, waiting times, retention rates per-intervention etc. I think this needs to be provided.

Response: We have modified language to better outline the randomization at the cluster level and the eligibility criteria at the individual level.

3. The baseline characteristics of the patients in the intervention and control groups differed in several characteristics which seems to imply that if they were truly randomly sampled then the clinic populations differed, again raising questions of recruitment bias.

Response: Thank you for this observation. All covariates across intervention and control populations were accounted for in final adjusted analyses.

4. Kaplan Meier curves should include the numbers of participants in the respective groups at each timepoint

Reponse: We undersdtand that risk tables might be useful however, we believe that the 95% confidence intervals are more appropriate.

5. Figure 5 is unclear. In the text it states that this is demonstrating MPR, however the legend on this plot is “Probability of making a visit (3 days) by intervention and refill interval”. This legend does not make sense grammatically or practically. Please consider amending the title and provide explanation of what this graphic shows.

Response: Thank you for pointing this out we have modified the language around figure 5 that we think resolves the confusion.

Results

1. Visit spacing is again unclear. I think you are saying that the appointments given were similar- median 91 days – although it seems the control group had quite a different IQR? Can you explain this given that all patients had a 90 day supply? Or is this to do with the introduction of MMD (see later question)? The next sentence states there was a significant difference in the “median time”. I don’t understand this- do you mean the actual time between visits or the appointment? Please make this clearer.

Response: Thank you for this note. We have re-visited the narrative in this secion of the results to improve clarity. Lines 273-275

2. The rest of this section is also unclear and seems to refer back to figure 5 which I think relates to this rather than the section where it is placed. Please consider rewriting this section to clarify what exactly is meant.

Response: Thank you, we have re-visited much of this section and revised much of the narrative in an effort to make more concise and clear. Lines 273-275

3. The study is described as “mixed methods” and while the team report on qualitative data collection including FGD and IDI they provide no information in the methods about analysis of qualitative data. Qualitative results are not presented as might be expected from a thorough analysis and so it is questionable what this adds to the paper. Whilst I am highly in favour of mixed methods research, I would strongly suggest that either more space is given to describe the analytical process and to present the results fully or to split the quantitative and qualitative methods and write each up more fully.

Response: Thank you for this observation. We have removed the qualitative data and results and plan to submit a separate manuscript with these data.

4. Are there any data available about time spent in the clinic, time of the staff to deliver the model, costs from the provider and patient perspective? These findings would strengthen the manuscript greatly.

Response: We do not have time and motion data for this model.

5. In the discussion the authors bring up multi-month scripting for the first time. If a change occurred mid-way through the intervention then this should be mentioned in the methods and possibly a timeline provided or some consideration of a sensitivity analysis pre and post changes. Was the MMD for longer than the 3 month dispensing being described in the methods?

Reponse: we have adjusted language to introduce multi-month scripting as sysnonymous with visit spacing for our purposes here. Lines 39 and 394

Limitations-

1. The greatest limitations are the undoubtable biases that will occur since this was not a randomised study. This should be discussed.

Response: The clusters were randomised and we believe this will address much of the concerns around biases.

Implications

1. I am not clear how the authors can state that the effects of FT went beyond those of MMD. As far as I can see these were not directly compared in this study. Suggest removing or revising.

Response: Figure five shows the break down of the effect of extended visit spacing/MMD and expedited clinic visits. We have revised language to make this conclusion clearer.

Data availability

1. Whilst I understand the sensitivities of this clinical data being available I think that additional data should be provided in the manuscript- such as the numbers requested to accompany Kaplan Meier curves and showing more of the actual numbers in tables.

Response: Thank you for this note. We have elected to include 95% confidence intervals to provide insight regarding the population at risk across the follow-up period.

Minor issues

Discussion

1. unfortunately lines are not numbered- paragraph 5 incomplete sentence “......95:95:95 targets is n sustaining....”

Response: Thank you, this has been addresses

2. Same para “FT....not being an elixir for all” this is strange phrasing consider rewriting

Response: Thank you, we have re-worded this sentence

Reviewer #2: It should be noted that the data is not publicly available without permission due to being MoH data and this is an acceptable exception.

The statistical rigor is exceptional for this manuscript - the qualitative methods need to be better described, particularly around coding, thematic analysis, and rigor of results.

There is also a need to straightforwardly convey the quantitative results - I found it difficult to parse the key results with the

acronyms and heavily described statistics. Best to lead with lay description and back-up with relevant statistics. I would not call it jargon, but felt a person needed to be fairly expert with these indicators to follow the thread.

Response: Thank you for your positive feedback and constructive criticism. We have removed the qualitative section and plan to submit a separate manuscript with these data.

This manuscript also fills a critical gap in the response and evidence on DSD and retention in care. Some minor edits would help to convey the context, standard of care in Zambia and how this changed over study duration, resource limitations of the intervention which restricted it to two intervention sites (infrastructure - i.e., space, which comes in late in the MS).

As far as qualitative results, it would also be helpful to describe why the pre-implementation FG findings are described, and also if they informed the intervention at all. The results write-up would benefit from presenting the qual findings longitudinally. The interspersion of quotes is especially effective in the later results paragraph, and would be good to interweave in the beginning qual paragraphs.

This is an excellent study and definitely fills a needed evidence gap in the HIV response, and casts a light on the difficulty of conducting implementation studies in vivo, in a dynamic policy and health system landscape.

End of comments:

We would like to thank the reviewers and editors, again, for assistance in improving this manuscript. We hope that it is a better fit for PLOS Global Public Health.

Kind Regards,

Jake

Jake M. Pry, PhD, MPH

Centre for Infectious Disease Research in Zambia (CIDRZ)

[jake.pry@cidrz.org](mailto:jake.pry@cidrz.org) | [jmpry@ucdavis.edu](mailto:jmpry@ucdavis.edu)

mobile: +1-936-661-6885 | +260 96 3088750
